# Dual-Self-Crosslinking Effect of Alginate-Di-Aldehyde with Natural and Synthetic Co-Polymers as Injectable In Situ-Forming Biodegradable Hydrogel

**DOI:** 10.3390/gels10100649

**Published:** 2024-10-11

**Authors:** Bushra Begum, Trideva Sastri Koduru, Syeda Noor Madni, Noor Fathima Anjum, Shanmuganathan Seetharaman, Balamuralidhara Veeranna, Vishal Kumar Gupta

**Affiliations:** 1 Department of Pharmaceutics, JSS College of Pharmacy, JSS Academy of Higher Education & Research, Sri Shivarathreeshwara Nagar, Mysuru 570015, India; bushrapharma2007@gmail.com (B.B.); trideva.k@gmail.com (T.S.K.); baligowda@jssuni.edu.in (B.V.); 2 Department of Pharmaceutics, Farooqia College of Pharmacy, Mysuru 570019, India; madni2865@gmail.com; 3 Department of Pharmaceutical Chemistry, Farooqia College of Pharmacy, Mysuru 570015, India; nfanjum79@gmail.com; 4 School of Pharmacy, Sri Balaji Vidyapeeth, Puducherry 607402, India; shanmuganathan@sbvu.ac.in

**Keywords:** hydrogel, ADA (alginate-di aldehyde), gelatin (GEL), oxidation, sodium metaperiodate/PEG (polyethene glycol)

## Abstract

Injectable, in situ-forming hydrogels, both biocompatible and biodegradable, have garnered significant attention in tissue engineering due to their potential for creating adaptable scaffolds. The adaptability of these hydrogels, made from natural proteins and polysaccharides, opens up a world of possibilities. In this study, sodium alginate was used to synthesize alginate di-aldehyde (ADA) through periodate oxidation, resulting in a lower molecular weight and reduced viscosity, with different degrees of oxidation (54% and 70%). The dual-crosslinking mechanism produced an injectable in situ hydrogel. Initially, physical crosslinking occurred between ADA and borax via borax complexation, followed by chemical crosslinking with gelatin through a Schiff’s base reaction, which takes place between the amino groups of gelatin and the aldehyde groups of ADA, without requiring an external crosslinking agent. The formation of Schiff’s base was confirmed by Fourier-transform infrared (FT-IR) spectroscopy. At the same time, the aldehyde groups in ADA were characterized using FT-IR, proton nuclear magnetic resonance (¹H NMR), and gel permeation chromatography (GPC), which determined its molecular weight. Furthermore, borax complexation was validated through boron-11 nuclear magnetic resonance (¹¹B NMR). The hydrogel formulation containing 70% ADA, polyethylene glycol (PEG), and 9% gelatin exhibited a decreased gelation time at physiological temperature, attributed to the increased gelatin content and higher degree of oxidation. Rheological analysis mirrored these findings, showing a correlation with gelation time. The swelling capacity was also enhanced due to the increased oxidation degree of PEG and the system’s elevated gelatin content and hydrophilicity. The hydrogel demonstrated an average pore size of 40–60 µm and a compressive strength of 376.80 kPa. The lower molecular weight and varied pH conditions influenced its degradation behavior. Notably, the hydrogel’s syringeability was deemed sufficient for practical applications, further enhancing its potential in tissue engineering. Given these properties, the 70% ADA/gelatin/PEG hydrogel is a promising candidate and a potential game-changer for injectable, self-crosslinking applications in tissue engineering. Its potential to revolutionize the field is inspiring and should motivate further exploration.

## 1. Introduction

Tissue engineering and regenerative medicine are promising new approaches developed as biological treatments for various diseases [1]. In this approach, injectable hydrogel, an important category of material, has been explored to achieve tissue repair and regeneration in the body, which probably mimics the extracellular matrix (ECM) of the tissue and thus has the potential to direct the migration, growth, and organization of cells during tissue regeneration [2,3]. Hydrogel, a three-dimensional crosslinked network polymer, allows for the holding of large amounts of water due to its hydrophilic structure. Thus, hydrogel networks can extensively swell in water media [4]. Hydrogels have several advantages over other biomaterial forms, including improved biocompatibility, flexibility, adjustable degradability, suitable mechanical strength, porous structure, and low cost [5,6]. Self-crosslinking, injectable in situ hydrogels have gained great attraction, as they are given parenterally via syringe, which is in sol form before administration and undergoes gelation once administered in the body under physiological temperature [7,8]. Self-crosslinking in situ hydrogels are easily delivered using a minimally invasive administration procedure, which improves patient comfort, and their crosslinks do not interfere with the injection procedure [9,10]. Various polymers and crosslinking techniques have already been studied to develop a self-crosslinkable gel-like substance appropriate for tissue engineering.

Consequently, natural polymers such as alginate, gelatin, and hyaluronic acid, and their combinations, are extremely promising [4]. Compared to synthetic scaffolds, natural scaffolds have a more significant effect on cell adhesion, migration, and division [11,12]. Alginate is a relatively abundant natural polysaccharide, and chemically, alginates are co-polymers mainly composed of two uronic acid residues: β-D-mannuronic acid (M) and ά-L- guluronic acid (G). One to four glycosidic bonds link these two acid units. Alginates have no regular structure but have residues as monomers. These monomers are mainly arranged in sequences of homopolymeric blocks (MM and GG blocks) and heteropolymeric blocks (MG or GM blocks). The monomer sequence distribution in the co-polymer gives rise to a flat ribbon-like structure for the MM blocks, a buckled ribbon-like structure for the GG blocks, and a helix-like structure for the MG or GM blocks [13,14,15,16]. Because of their high viscosity in aqueous solutions, good stability, gel ability, and biocompatibility, alginates are widely used in tissue engineering [17]. There are still specific concerns with this structure, as alginates are non-biodegradable, provoke foreign body cell reactions, and can evoke an immune response [18,19,20]. Alginate cannot degrade in mammals, due to a lack of alginate-degrading enzymes, and high-molecular-weight alginate restrains renal clearance. To overcome these limitations, alginates are chemically modified by periodate oxidation [21,22]. This oxidation process was carried out in an ethanol/water dispersion or an aqueous medium system using sodium metaperiodate (NaIO4) as an oxidizing agent in the dark [23,24]. The oxidized alginate has more reactive groups, facilitating quicker degradation [25]. These oxidized alginates crosslink with other natural polysaccharides, such as gelatin chitosan, and are used in cartilage, bone regeneration, and drug delivery. Collagen undergoes hydrolytic degradation to produce a natural protein called gelatin, which is water-soluble and biodegradable. Gelatin is highly biocompatible and biodegradable, has an affinity for cells, has low antigenicity, and promotes cell adhesion, migration, and proliferation [26,27,28,29,30]. Adding borax into ADA and gelatin enhances the gelation time. Borax has also been used for medical purposes for a very long time in humans [31,32].

Additionally, borate treatment has a long history of usage in treating arthritis; reports have been published about the immunomodulatory effects of calcium-fructoborate in rats with arthritis [26]. However, gelatin’s weak mechanical property reduces its usage; this can be solved by crosslinking with ADA [33]. PEG (polyethylene glycol) is a synthetic polymer. It is used in tissue engineering, medication delivery, cell encapsulation, and other biological applications. The system’s mechanical properties have been improved by reinforcing natural polymers with synthetic ones [34]. The objective of this research was to develop biodegradable, injectable, in situ, dual-crosslinked hydrogels forming a composition of alginate di-aldehyde, gelatin, and PEG in the presence of 0.1 M borax without using any extraneous crosslinking agents. In this study, we examined the gelation time, gelation temperature, swelling ratio, and degree of crosslinking of hydrogels prepared by borax complexation, and Schiff’s reaction of ADA/gelatin/borax, morphology, degradability, compressive properties, injectability, and rheology were analyzed.

## 2. Results and Discussion

### 2.1. Oxidation

**Figure 1:**
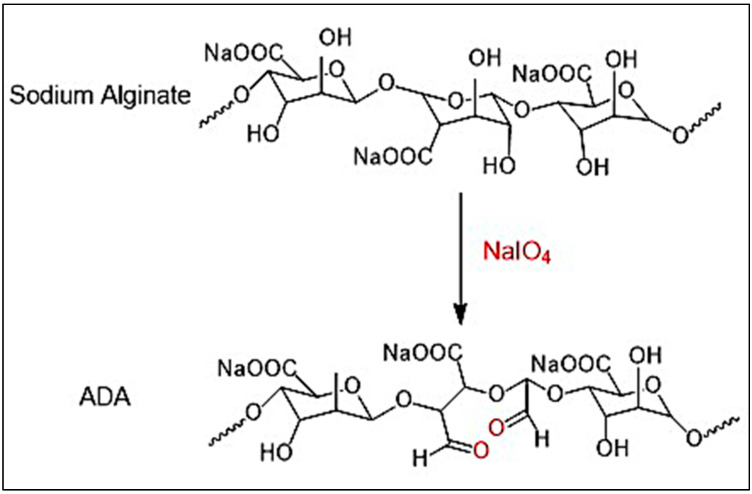
Alginate-di-aldehyde (ADA) was synthesized by oxidation reaction from sodium alginate using sodium metaperiodate as an oxidizing agent in the ethanol–water system for 6 h in the dark at room temperature.

**Figure 2:**
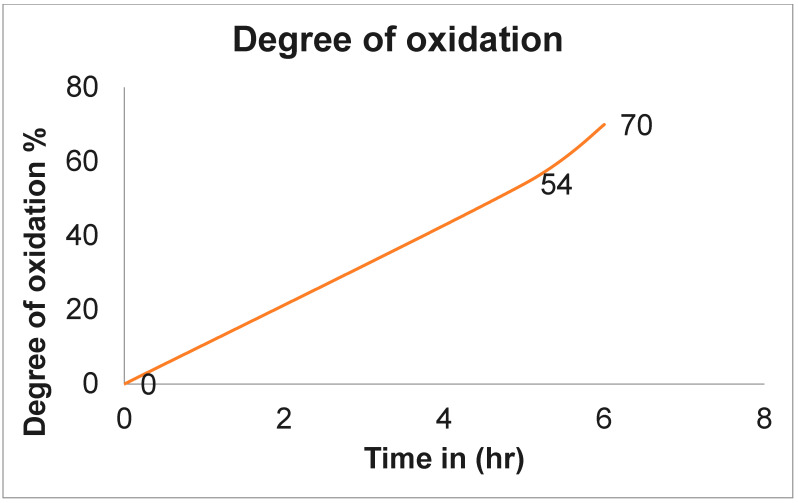
The influence of reaction time on the oxidized degree of alginate: 54% ADA-5 h, 70% ADA-6 h.

The natural polymer alginate is challenging to work with and produces extremely viscous solutions even at low concentrations. It is difficult to handle and may limit its use in drug delivery [26,35]. However, in this work, we investigated the response in a heterogeneous medium in a smooth and more accessible manner using the dispersion of an ethanol–water system. This medium made preparing a considerable amount of oxidized product with less solvent easy, simplifying the reaction. Using this medium, sodium alginate was oxidized in the presence of an oxidizing agent, such as sodium metaperiodate (NaIO_4_), at room temperature for 6 h in the dark. Since periodate is naturally unstable, it degrades over time to produce radicals, mainly when light is present. Thus, oxidations are preferably performed with freshly made periodate solutions without light, which enhances the degradation properties. The repetitive unit of the alginate chain consists of two OH groups at the C-2 and C-3 positions and one COOH group at the C-6 position, and selective modification of the C-2 and C-3 positions with the OH group is challenging due to the minor reactivity difference in the functional group types [35]. Using NaIO_4_ leads to the cleavage of C-2 and C-3 bonds and introduces aldehyde groups at the C-2 and C-3 positions, as shown in Figure 1 This ring opening has a substantial effect on the chain extension with a new reactive group along the backbone, which strictly destroyed the rigid structure and gave a more flexible polymer (ADA) [36,37]. The current work shows that sodium alginate was oxidized to the extent of (54%, 70%). The oxidized uronic acid group in the alginate was determined by the consumption of varying amounts of NaIO_4_ with the obtained yield, as shown in Table 1, and the influence of reaction time on the degree of oxidation of alginate is shown in Figure 2. FT-IR spectra of alginate and the formation of ADA are shown.

### 2.2. Molecular Weight by Gas Permeation Chromatography (GPC)

**Figure 3:**
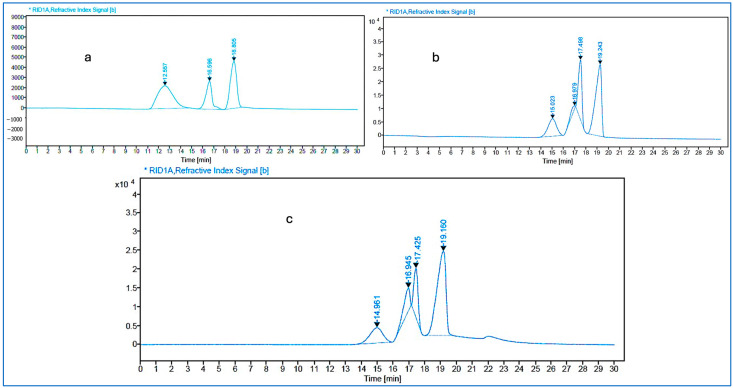
GPC Chromatograms of (**a**) sodium alginate and (**b**) ADA 70% and (**c**) ADA54% the GPC chromatograms represent a significant difference when compared with sodium alginate, 70% ADA, and 54% ADA (*p* < 0.05).

The molecular weights (Mws) of sodium alginate and ADA were measured using GPC. Mw practically impacts the hydrogels’ stability, degradation, and mechanical properties [36]. The molecular weight of each sample was determined to analyze the amount of polymer cleavage from the periodate oxidation. After oxidation, a substantial decrease in molecular weight occurs, attributed to the extensive cleavage of the polysaccharide chains. Moreover, as the dosage of sodium periodates increases, a gradual reduction in molecular weight indicates that the degree of oxidation was increased. Low-molecular-weight alginates have a high chance of finding the correct crosslink functional groups because they can pierce gelatin chitosan chains more quickly than equivalents. The polymer’s molecular weight also influences the intrinsic viscosity value of the polymer. With oxidation, the viscosity and subsequent molecular weight of alginate decreased significantly. As unmodified alginate with high molecular weight is difficult to degrade and clear out of the body through the kidney, alginate with low molecular weight degrades faster [38,39]. In this work, the molecular weight (Mw and Mn), polydispersity (Mw/Mn), and retention times of the masses of (a) sodium alginate, (b) ADA (54%), and (c) ADA (70%) are shown in Table 2 and Figure 3. As retention time increases, molecular weight decreases, increasing the potential for chain scission. A polydispersity close to 1 defines the characteristic of the product polymer. Alginate dialdehyde (70%) has a much lower molecular weight than alginate di-aldehyde (54%) sodium alginate and has a statistically significant difference (*p* < 0.05).

### 2.3. Viscosity

In Table 3, it is demonstrated that the viscosity of ADA is significantly reduced when oxidized by sodium metaperiodate in the ethanol–water system. This is because ADA’s molecular weight is lower than alginate’s. At a 1 g/500 mL concentration, the intrinsic viscosity of ADA 70% is significantly lower than those of sodium alginate and ADA 54%. However, as the degree of oxidation increases, the molecular weight reduces, decreasing the viscosity. This is partially attributed to increased entire-chain flexibility and inter-chain crosslinking with gelatin [37,40,41,42]. Further studies were conducted to validate this theory, as shown in the following sections.

### 2.4. NMR Analysis

**Figure 4:**
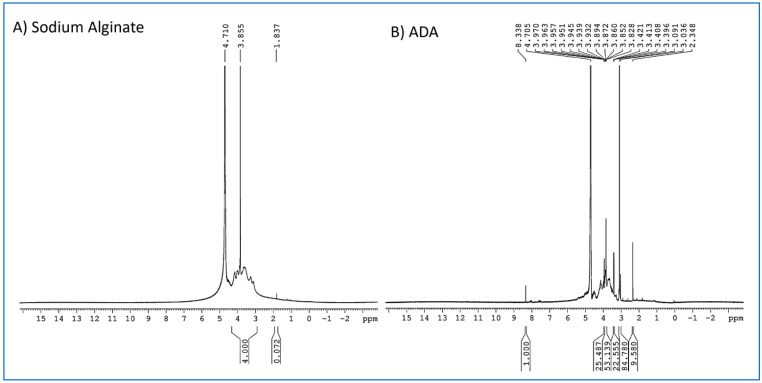
NMR spectra of (**A**) alginate and (**B**) ADA.

The results of ^1^H NMR spectroscopy of alginate and ADA are shown in Figure 4. In Figure 4A, sodium alginate exhibits peaks ranging from 3.855 ppm to 4.710 ppm, which belong to protons of G and M units. The signals appearing at 3.855 ppm correspond to H_2_-G and decrease and move to the high field for cleavage of the C2–C3 bond of the uronic acid monomer, and the signal at 4.710 ppm corresponds to H_5_-G. After the oxidation of alginate to ADA, the signals observed at 3.828 ppm and 4.705 ppm are assigned to H_3_-M and H_5_-G and the one new peak at 8.338 ppm is assigned to CHO. The above changes all confirm the formation of ADA.

### 2.5. Degree of Crosslinking

**Figure 5:**
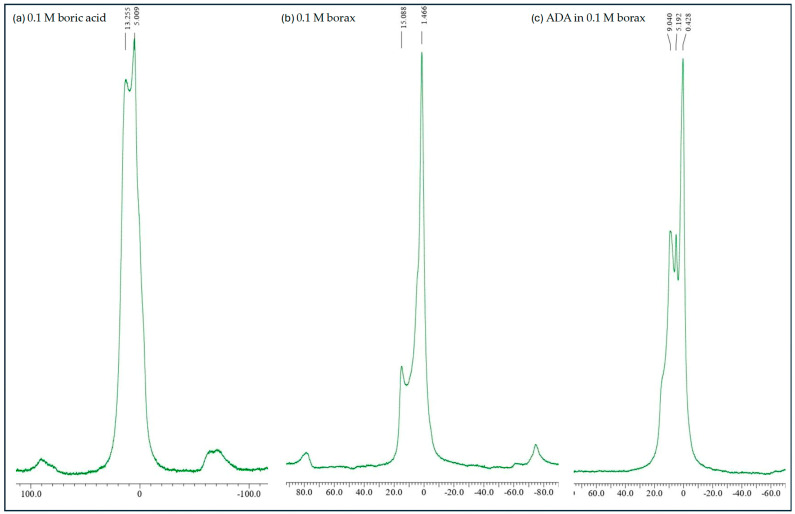
^11^B NMR spectra of (**a**) 0.1 M boric acid, (**b**) 0.1 M borax, and (**c**) ADA in 0.1 M borax.

**Figure 6:**
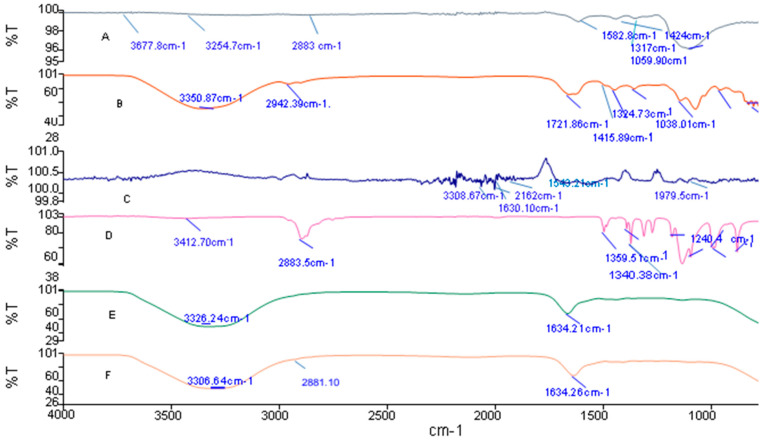
FTIR spectra of (**A**) sodium alginate, (**B**) alginate di-aldehyde, (**C**) gelatin, (**D**) PEG, and (**E**) ADA + GEL and (**F**) ADA + GEL + PEG. (The bands are in representations of cm^−1^).

Crosslinking the aldehyde groups of oxidized alginate with divalent and trivalent cations, such as Ca^2+^, Ba^2+^, Sr^2+^, Fe^3+^, and Al^3+^, results in weak physical interactions forming physical hydrogels [43]. Furthermore, a chemical hydrogel can be obtained from Schiff’s base reaction (C=N) through the interaction between amino groups of lysine or hydroxyl lysine side-groups of gelatin and the aldehyde group of ADA in the presence of borax, as shown in Figure 5. The degree of crosslinking of hydrogels was determined using the ninhydrin assay, and the results are listed in Table 4. According to the results, A2 offered a higher crosslinking degree due to the availability of aldehyde groups of ADA to amine groups of gelatin, representing a higher crosslinking degree than A1. This Schiff’s base reaction in the presence of borax enhanced the gelation property. FT-IR spectra confirmed the mechanism of Schiff’s response, as shown in Figure 6, where a new band emerged at 1634.21 cm^−1^ assigned to the CN double-bond.

### 2.6. Fourier-Transform Infrared Spectroscopy (FT-IR)

In Figure 6A, the FTIR spectrum of alginate shows two strong bands, appearing at 1582.8 cm^−1^ and 1424 cm^−1^, which are attributed to asymmetric and symmetric stretching vibrations of –COO– groups on the polymer backbone. This figure also shows the C-O single-bond stretching vibration at 1317 cm^−1^. In addition, there are the polysaccharide-specific bands at 1059.90 cm^−1^ of the C–O–C (cyclic ether) stretching vibration, the bands at 2883 cm^−1^ of C–H stretching, the broadband due to the hydrogen bond, and OH groups between 3254.7 and 3677.8 cm^−1^ attributed to the complex vibrational stretching, associated with free inter- and intra-molecular bond hydroxyls. Figure 6B, showing the spectra of ADA, show a shifting of a C-O single-bond peak from 1317 cm^−1^ to 1324.73 cm^−1^, resulting in chain cleavage.

Moreover, the C–O–C (cyclic ether) band at 1038.01 cm-^1^ is reduced remarkably due to the chain’s cleavage. The new band at 1721.86 cm-^1^ in the oxidized sample indicates the presence of an aldehyde group, which suggests that the oxidation reaction had occurred. The broad characteristic band at 3350.87 cm^−1^ is associated with the –OH stretching vibration. The stretching vibration of the H-C aldehyde can be seen around 2942.39 cm^−1^. Figure 6C for gelatin shows the characteristic absorption bands of its protein structure. The bands at 1630.10 cm^−1^ and 1543.21 cm^−1^ are assigned to the N–H stretching vibration peaks for amide I and II, respectively. Figure 6D for PEG shows that the characteristic absorption bands at 1240.4 cm^−1^ and 2883.5 cm^−1^ are attributed to the bending and stretching vibrations of C-H, respectively. In Figure 6E, showing the spectrum of ADA-GEL-borax crosslinked hydrogel, the aldehyde group band of ADA at 1721.86 cm^−1^ vanishes, and a new band emerges at 1634.21 cm^−1^ assigned to a CN double-bond. The new band is because of the Schiff’s base reaction within the amine group of gelatin and the aldehyde group of ADA. Figure 6F for ADA/GEL/PEG shows that the PEG characteristic absorption bands at 2883.38 are stronger and show strong evidence of the semi-IPN interactions.

### 2.7. ADA–Borate Interaction

There are many reports that the gel formation is due to the ability of borax to complex with OH-group-containing compounds such as poly(vinyl alcohol), poly (glyceryl methacrylate), and polysaccharide. Borax dissociates to an equal amount of boric acid and borate in an aqueous medium, as shown below [26].

(1) $$B_{4}O_{7}^{2-}+7H_{2}O⇋2B{\left(OH\right)}_{3}+2B{\left(OH\right)}_{4}^{-}$$

**Figure 7:**
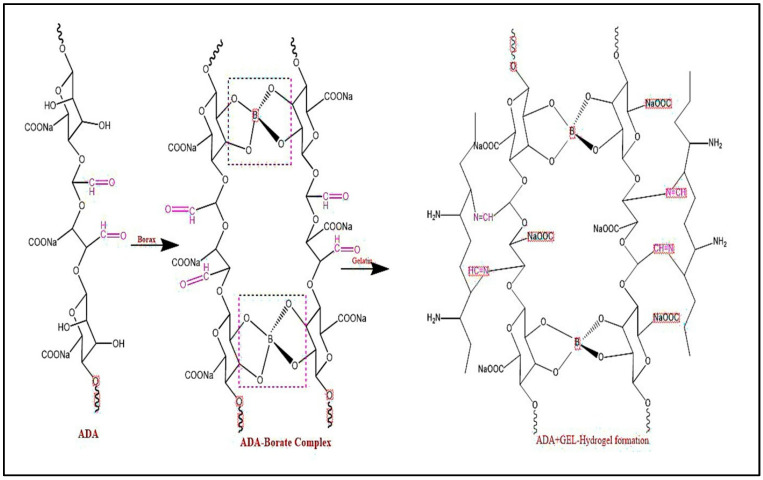
Formation of ADA-gelatin hydrogel in the presence of borax.

Borax treats osteoporosis and inflammatory diseases like joint pain and arthritis. When the backbones (dialdehyde) dissolve in borax, the OH groups on the dialdehyde and the tetra hydroxyl borate OH groups form a hydrogen bond. Thus, the OH of ADA is a ligand for the complexation of borates, leading to mono/di-complexes [44,45]. ^11^B NMR spectroscopy is a well-established tool for studying the structures of borate–ligand complexes. This method relies on the principle that the interconversion of the borate–ligand complexes is slow enough to detect discrete complexes without excessive lifetime broadening or signal averaging. Figure 7 shows the formation of ADA-gelatin hydrogel in the presence of borax. The ^11^B NMR spectra of 0.1 M boric, 0.1 M borax, and ADA + 0.1 M borax. The peaks at δ13.22 and 5.009 ppm are characteristics of boric acid; 5.19 ppm is also seen in ADA+ 0.1 M borax. The chemical shift at δ values of δ15.44 and 1.466 ppm is attributed to an uncompleted borate signal of 0.1 M borax. The case of ADA in borax, along with the 5.19 ppm peak, additional peaks can be seen at δ 9.0 ppm, which can be due to the complexation of borate with diols of ADA leading to the formation of mono- and di-complexes. The reason for the rapid gelation of ADA with gelatin in the presence of borax can be attributed to its role in promoting further crosslinking between the ADA’s aldehyde groups and the amino groups of the gelatin’s lysine residues via Schiff’s base formation. This process leads to a stage where nearly all of the polymer chains are linked to other chains at multiple points, resulting in one massive covalently bonded molecule at the gel point [46,47,48]. This shows that the formation of an in situ gelling, self-crosslinked system occurs as a result of dual-crosslinking methods, which involve first physically crosslinking ADA chains with borax (borax complexation) and then chemically crosslinking with gelatin (Schiff’s reaction) all without the need of crosslinking agents.

Furthermore, borax enhanced the solubility of ADA in an aqueous medium, which increased as the borax concentration ascended to 0.1 M. The solubility decreased by half at 0.05 M borax, while a 7% solution of ADA was readily prepared at 0.1 M borax. A higher degree of oxidation was appropriate with 0.1 M borax. 

### 2.8. Estimating the Gelation Temperature (T) and Gelation Time (t)

**Figure 8:**
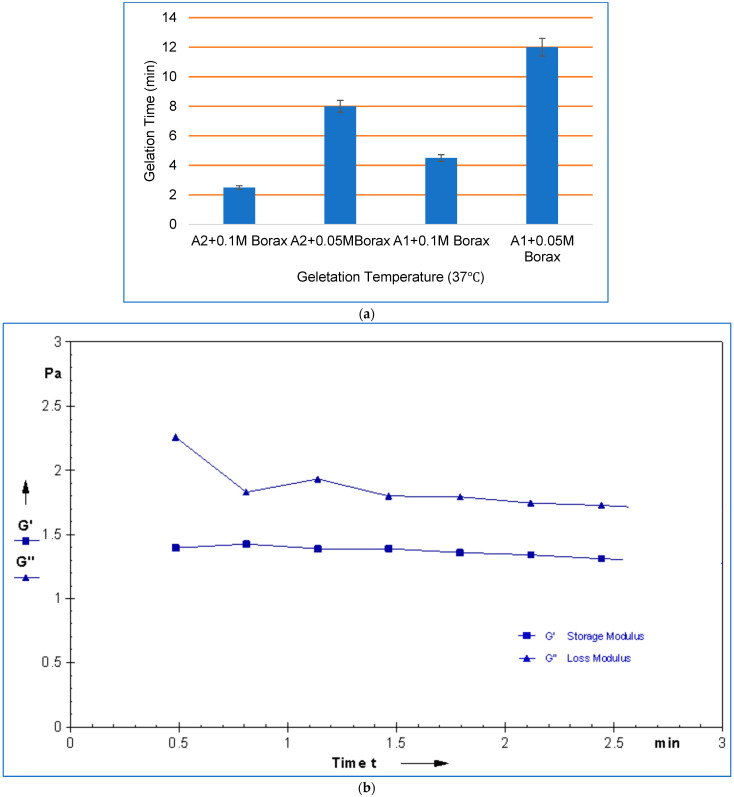
(**a**) Gelling time and gelling temperature of hydrogels. (**b**) Rheological property analysis of storage moduli (G)′ and loss moduli (G)″ of 70% ADA + GEL + PEG as a function of time after mixing AD and gelatin under constant shear rate at 25 °C.

For injectable hydrogel intended for clinical application, gelation temperature (T) and gelation time (t) are essential characteristics. Gelation time: the time required for the gelatin and ADA to crosslink [49,50]. Injectable hydrogels must be in liquid form at the time of injection, but they should transform into a gel after injection [51]. In this work, using the magnetic stirrer method, gelation temperature and gelling time were determined at different temperatures for formulated hydrogels with varying concentrations of borax, as shown in Table 5. No gelation was observed when gelatin and ADA were dissolved in water. As shown in Figure 8a, in the presence of 0.1 M borax, the gel was formed in 2.5 min and 8 min with 0.05 M borax for A2 hydrogel at 37 °C and there was no gelation at 25 °C and 30 °C. For A1 hydrogel, it was 4.5 min and 12 min for 0.05 M borax at 37 °C and there was no gelation at 25 °C and 30 °C. Gelling time can be adjusted; it decreases with 0.1 M borax concentration, with an increase in gelatin content, and with a high degree of oxidation, which made the aldehyde group react with amino group of gelatins to form a chemically crosslinking network structure via Schiff’s reaction. It is noticeable how the concentration of borax affects the gelling time. We suspect that this is because the alkaline pH of the medium makes it easier for Schiff’s base to develop and for borax to interact with the hydroxyl group of the polysaccharide, thereby accelerating the gelation process, refs. [37,40]—a low concentration of borax of 0.05 M results in a lack of gelation.

Fast gelation times for injectable systems are crucial since they will make filling the defect site easier. Time sweep rheological analysis, as shown in Figure 8b, was carried out to confirm the gelation time of the developed crosslinked hydrogel at 37 °C, where the storage modulus (G′) and loss modulus (G″) corresponding to the elastic and viscous components, respectively, were monitored concerning time to determine the rate and extent of crosslinking [49,52]. The elastic modulus G′ is less than the loss modulus G″ before gelation, exhibiting a fluid-like performance and predominately viscous appearance at the start of the gelation. Compared to G″, the G rate increases more quickly. One crossover point of G′ and G″, where G′ is equal to G″, is defined as the state of gel formation [53]. For A2 hydrogel, the crossover points of G and G″ were at 2.5 min, and the time needed to combine the gelatin and ADA to initiate the experiments was 1 min. As a result, it took roughly 3.5 min to reach the crossover point, near the value noted during the gelation time.

### 2.9. Swelling

**Figure 9:**
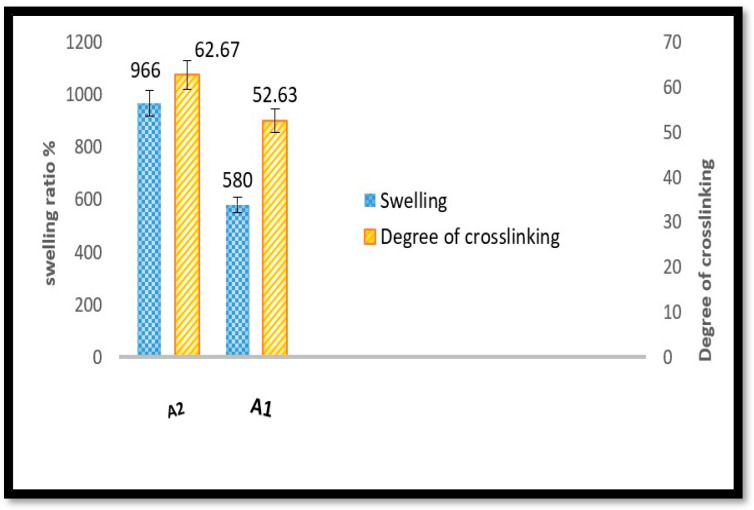
Swelling ratio and degree of crosslinking of A1 and A2 hydrogels.

The extension of crosslinking in hydrogel networks is commonly accessed by swelling measures. Hydrogels are synthesized when polymer chains physically or chemically link together; a specific amount of solvent is absorbed by the polymer, causing it to swell without breaking down the crosslinking structure of the polymer. This unique property enables the hydrogel to retain its shape and structure even when exposed to water, making it an essential biomaterial. The water content and holding capacity with swelling properties promote cellular growth and ECM deposition [1,54]. A biomaterial’s ability to transfer nutrients and metabolites to cells and absorb bodily fluids between the cells and the medium results in the swelling capacity of the hydrogel, respectively [53,55]. In this work, the water uptake in the hydrogel of A1 and A2 samples was carried out in PBS (phosphate-buffered saline) at pH 7.4 at 37 °C. There was an increase in swelling and a maximum of up to 24 h, which remained constant for four weeks and slowly started degrading. As shown in Table 6 and Figure 9, this increase in swelling was due to an increase in the degree of oxidation in the presence of a high content of gelatin and PEG. A higher degree of oxidation of A2 hydrogel results in more aldehyde-crosslinkable groups combined with gelatin, leading to more efficient network formation.

On the other hand, insufficient aldehyde groups for crosslinking in the hydrogels with a low degree of oxidation of A1 hydrogel lead to partial network formation. This could have led to the breakdown of uncrosslinked gelatin and decreased water retention. PEG chains have a dynamic attachment to a cloud of water molecules, minimizing the extracellular matrix’s high water content. Due to this attachment, PEGs are categorized as highly hydrophilic polymers. This leads to a gel that swells with a significant increase in fluid uptake [48,56,57]. PEG hydrogel surroundings are often permissive, facilitate nutrition transport easily, and keep hydrophilic biomolecules bioactive [58]. The swelling ratio of A2 was a covalently crosslinked hydrogel and was mechanically stable in its fully hydrated state in PBS without dissolving when immersed in it, as assessed by handling. This is attributed to the high porosity and hydrophilicity of the hydrogels compared to A1. There was a satisfied statistical difference (*p* < 0.05) for swelling for A2 hydrogel.

### 2.10. Degradation Profiles

**Figure 10:**
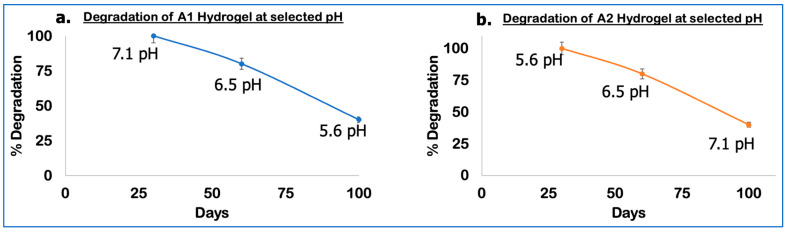
(**a**) Degradation of A1 hydrogel and (**b**) A2 hydrogel at 37 °C for different days and different pHs.

For a hydrogel to be effective in prospective biological applications, it must degrade. The hydrogel’s relative degradability facilitates cell diffusion nutrition flow and provides enough space for cell growth and tissue formation [5,46,59,60]. Alginates are not broken down in mammals, as they have a significant molecular weight that causes a sluggish renal clearance from the body; in contrast, ADA showed significant biodegradability due to its low molecular weight. PEGs shield the polymers from unwanted interactions because of their inert state. During matrix degradation, PEG maintains ECM deposition and encapsulates cell viability. PEG also degrades significantly [61]. In this study, hydrogel degradation (A1 and A2) was analyzed as an outcome of incubation time in PBS buffer solution at different pHs at 37 °C; the degradability of the gels is portrayed in Figure 10. In 10 days, up to 80% of A1 hydrogel degraded at pH 7.1, up to 50% in 60 days at pH 6.5, and up to 20% in 100 days at pH 5.6. In 100 days, up to 40% of A2 hydrogel degraded at pH 7.1, up to 80% at pH 6.5, and up to 100% at pH 5.6 in 30 days. All samples showed a significant difference of (*p* < 0.05). The following two steps can be used to characterize the degradation process. The hydrogel network did not break at first; only a few crosslinks were broken, which led to an increase in the network’s lattice size and a significant increase in water absorbance. Following this, the hydrogel was lost due to the dissociation of the entire crosslinking network and the broken crosslinked bonds reaching a critical value [62]. Thus, degradation can be controlled by adjusting the polymer Mw and composition. By dual-crosslinking, the covalently linked hydrogels result in prolonged degradation due to a higher degree of oxidation exhibiting a lower molecular weight compared to alginates; the in vitro hydrogel degradation helps investigate the structure–function interactions of the hydrogel and forecast possible applications [61,62].

### 2.11. Mechanical Property

**Figure 11:**
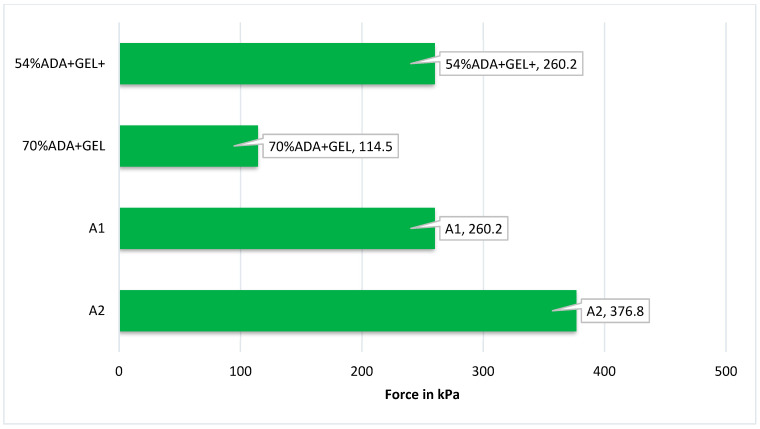
The compressive stress–strain curves of A2, A1, 70% ADA + GEL, and 54% ADA + GEL. When force was applied, there was a significant difference (*p* < 0.05).

**Figure 12:**
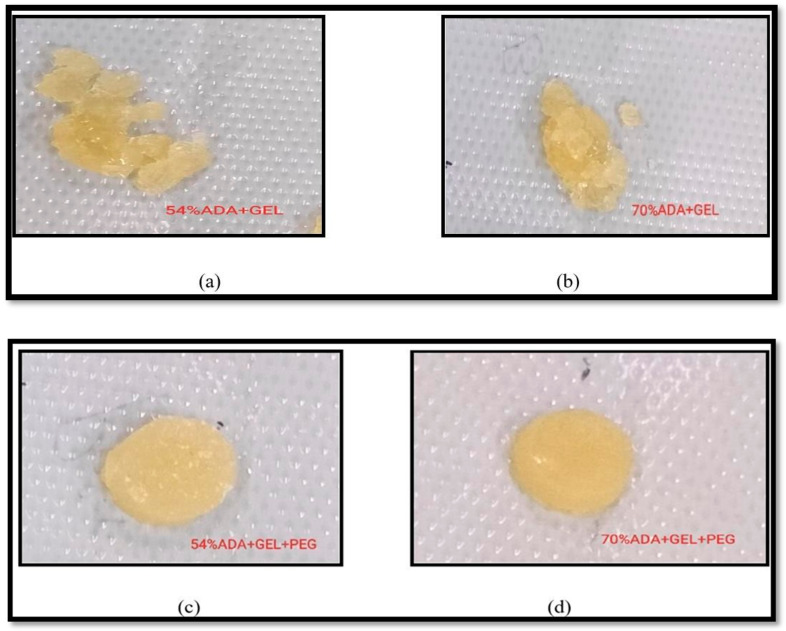
Photographs of hydrogels with the compressive strains of 80%: (**a**) photograph of (70%) ADA + GEL completely crushed, (**b**) (54%) ADA + GEL completely crushed, (**c**) 54% ADA + GEL + PEG with reduced recoverability, and (**d**) 70% ADA + GEL + PEG with good recoverability.

Gels designed for use as implants must possess sufficient mechanical strength to sustain loads or hold onto their physical characteristics until the gel’s intended purpose is achieved or newly formed tissue replaces them [63]. Hydrogel networks possess an enhanced mechanical capacity because they can evenly distribute external forces through discrete crosslinking points and other factors such as the degree of oxidation (modification of chain), composition, molecular weight, and reaction conditions during hydrogel formation [64,65]. The brittleness of the gel decreased when low-molecular-weight oxidized polymer chains were alone (ADA) in solution. This is due to a decrease in the physical interaction between the chains. Yet, the mechanical stability of the gels can be enhanced by the addition of a second polymer, such as gelatin, along with a lower-molecular-weight oxidized product, resulting in the coupling of the two polymer chains, by Schiff’s base reaction, leading to hydrogel formation [56,64]. As shown in Figure 11, the compressive strength curve of the hydrogel of A2 hydrogel was increased to 376.8 kPa compared to 70% ADA + GEL (114.5 kPa), and similarly, A1 hydrogel was 260.2 kPa compared to 54% ADA + GEL (260.2 kPa). The hydrogel’s pore walls lowered as the static or dynamic load gradually increased. The hydrogel’s pore walls then began to collapse progressively under increasing stress. A covalently crosslinked hydrogel of 70% ADA + GEL showed minimal impact on compressive strength, which was maintained due to increased oxidation. These weak mechanical properties of natural polysaccharides can be overcome by incorporating PEG, a synthetic polymer that interpenetrates and enhances hydrogel’s mechanical properties and hydrophilicity. We can facilitate the co-polymerization of two polysaccharides, thereby developing entanglement between synthetic and natural polymers. This interpenetration helped overcome the mechanical fragility of conventional hydrogel and its hydrophilic nature in the absorbing fluid by showing gelling properties [66]. Thus, the results show that the mechanical strength was enhanced because of increased oxidation, and the addition of PEG (A2 hydrogel) increased the crosslinking between the two polymer chains. The photographs of hydrogel when force was applied are shown in Figure 12.

### 2.12. Morphological Studies

**Figure 13:**
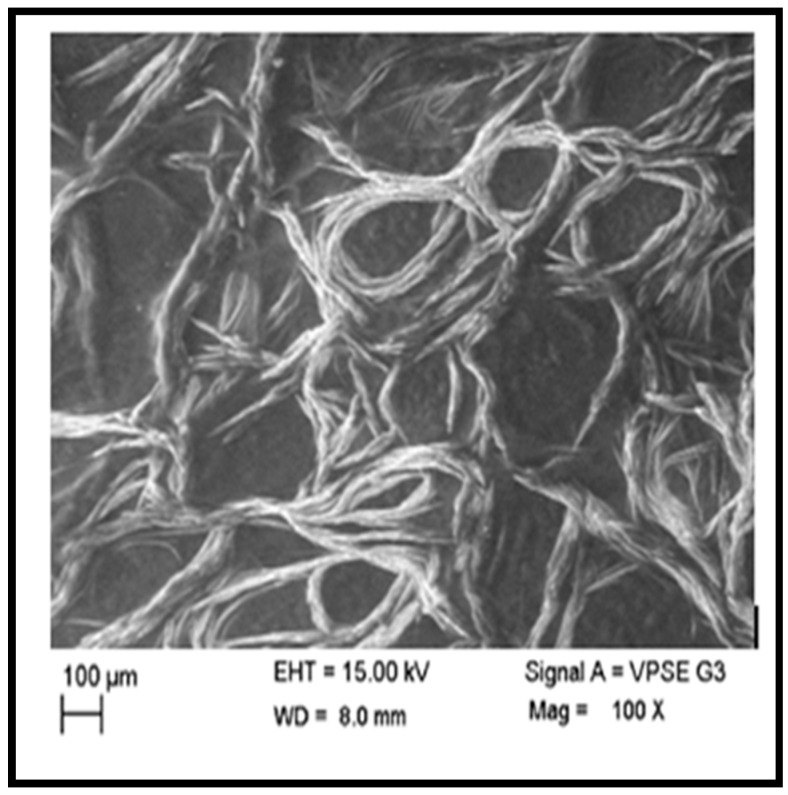
SEM images of 70% ADA + GEL + PEG hydrogel.

For cell development, migration, elimination of waste, and signaling, the ECM (extracellular matrix) is a heterogeneous complex that provides structural support, where hydrogel mimics the property of ECM [67]. The pore size affects the orientation and arrangement of cells in hydrogels throughout their later growth phases. The porosity of hydrogels is determined by the total volume of pores found inside them. Pore sizes ranging from 10 to 100 µm suggest fibrous tissue or unmineralized osteoid [67,68]. Comprehensive research was conducted on the hydrogel composition of A2 hydrogel since this hydrogel was the ideal composition for molecular weight, compressive strength, gelling time, and swelling ratio. An SEM (scanning electron microscopy) image of A2 hydrogel was analyzed, as shown in Figure 13. A2 hydrogel appears to be less compact and sticking; this dispersion degree of hydrogel is due to its reduced molecular weight, increased degree of oxidation, and the influence of crosslinking. This hydrogel system showed a highly porous and channelized structure with random branching. The channels were not continuous and were divided into multiple segments by thin separators.

Conversely, adding PEG allowed the hydrogel’s volume to expand, possibly due to the PEG’s “spacer effect” intercalation between the ADA chains and gelatin, exhibiting good compatibility. Hydrogel showed a porous structure with an average pore size distribution from 12 to 100 µm, similar to other hydrogel macromolecules. The pore size lay between 40 and 60 µm in the case of A2 hydrogel. This outcome was comparable to the PVA hydrogel results that were previously published [69].

### 2.13. Syringeability

The viscosity value influences syringeability; as the viscosity value increases, syringeability decreases; the syringeability values for hydrogel are shown in Table 7. The values were between 276 s for A1 hydrogel and 212 s for A2 hydrogel. This difference was significant (*p* < 0.05) for both hydrogel formulations. The injection force measured with an 18-gauge needle was 0.1 N for both hydrogels; the alginate’s viscosity is related to its molecular weight and typically increases with an increase in molecular weight [69]. ADA has a low molecular weight, so viscosity was low as both hydrogels were easily injectable through the needle. A decrease in the intrinsic viscosity was found in 70% ADA, although the required time to expel 1 mL of ADA 70% was lower than AO 54%.

## 3. Conclusions

This research has shown that a dual-self-crosslinking, biodegradable, in situ-forming injectable hydrogel was developed. The results showed that the degree of oxidation (ADA) was increased with the increase in oxidizing agent sodium metaperiodate with a reaction time of 6 h in an ethanol–water system. With the rise in the degree of oxidation, the molecular weight was reduced, decreasing the viscosity. ADA and gelatin were formulated using borax by borate complexation and Schiff’s base reaction. Hydrogel gelation time was reduced by increasing the borax concentration and using a high degree of oxidation and gelatin content. Degradability was significant depending on the lower molecular weight. The swelling of hydrogel was improved with a higher degree of oxidation and a high content of gelatin. Compressive strength was enhanced because of the presence of PEG, and syringeability was controlled. SEM images showed a porous and channeled structure suitable for the proliferation of cells, nutrient transfusion, and release of growth factors on the hydrogel matrix. The final formulation chosen was 70% ADA + GEL + PEG as a high-quality hydrogel for the self-crosslinking injectable scaffold for tissue engineering.

## 4. Materials and Methods

### 4.1. Materials

Sodium alginate (SA) (medium-viscosity-grade, viscosity of 2%, solution of 3500 cps at 25 °C, (Nice Chemical Pvt Ltd., Cochin, India); gelatin (Bloom 300, Type A, porcine skin); sodium metaperiodate (Lobia Chemie, Mumbai, India); borax (Alpha Chemika, Mumbai, India); dialysis tubing, MW = 14,000 (obtained from Hi media labs, Maharashtra, India); and ninhydrin reagent (Meru cam Pvt Ltd. Maharashtra, India, PEG) were obtained from (J.B.Chemicals & Pharmaceuticals Ltd., Gujarat, India). All the chemicals used in this study had a purity level greater than 95%.

### 4.2. Oxidation Reaction on Sodium Alginate

An amount of 20 g of sodium alginate was suspended in 100 mL of ethanol. Then, a different sodium metaperiodate was dissolved in 100 mL of deionized (DI) water. The solution was then slowly added to the sodium alginate dispersion to obtain different percent-oxidized alginates. This was stirred magnetically in the dark for 6 h. Next, the content was dialyzed against distilled water (2.5 L) using a dialysis tube (MW-14,000), lyophilized, and stored in a desiccator [70].

### 4.3. Determination of Oxidation Degree by UV–Visible Absorption Spectroscopy

The degree of oxidation was determined by measuring the unconsumed sodium metaperiodates with the starch indicator using ultraviolet (UV) spectroscopy. Equal quantities of 20% KI (potassium iodide) and 1% w/v starch solution were combined in a phosphate buffer (pH 7) solvent to produce the indicator solution. An amount of 250 mL of deionized water (DI) was added to 1 mL of alginate di-aldehyde solution. Then, 1.5 mL of the indicator solution was mixed with 3 mL of the diluted alginate di-aldehyde solution. Next, using a UV spectrophotometer (UV-Vis spectrophotometer 117 (1 NM) Gujarat (India)), the absorbance of the tri-iodine starch combination was determined at 486 nm. The periodate concentration in the sample was determined by utilizing the molar absorption coefficient previously determined from the complex’s absorbance versus the concentration of IO4. When IO4 interacted with sodium alginate, the difference between the initial and final concentrations of the compound oxidized the appropriate hydroxyl groups, causing them to change into aldehyde groups [49].

### 4.4. Determination of Molecular Weight

Using a GPC Agilent 1260 Infinity II with IR detectors, gel permeation chromatography (GPC) was used to estimate sodium alginate’s and oxidized alginates’ molecular weights. A 10 mL aqueous sample solution (10 mg/mL) was filtered using a 0.22 µm pore nylon syringe filter membrane to eliminate dust particles. The injection volume of the sample was 20 µL. The eluent was 200 ppm of sodium azide in a water sample at a flow of 1 mL/min under an elution temperature of 30 °C. Before measurements, the apparatus was calibrated using the PEG, PEO 50 standard (CAS Number:25322-68-3) (Sigma-Aldrich, Wien, Austria) [40,41].

### 4.5. Viscosity Measurement

Using a 2 g/500 mL volume of sample, a viscometer Model RVDLII1Pro (AMETEK Brookfield, PA, USA) was used to measure the viscosity of the ADA and alginate solutions [41,42].

### 4.6. NMR Analysis (Nuclear Magnetic Resonance)

Using ^1^H NMR spectroscopy, SA and ADA spectra were recorded in deuterium oxide at 50 °C using a Bruker AV 400 spectrometer (Bruker, Billerica, MA, USA) [49].

## 5. Preparation of Hydrogel

As per Table 8, hydrogel synthesis was carried out using the following method: Firstly, ADA was dissolved in borax; then, gelatin was dissolved in deionized water at room temperature using a magnetic stirrer at 300 rpm. After the alginate solution was dissolved entirely, gelatin solution was added. PEG was added to the mixture and stirred again [42,49].

### 5.1. Hydrogel Characterization

#### 5.1.1. Determination of the Degree of Crosslinking

The degree of crosslinking of hydrogels was ascertained using the ninhydrin assay. This assay was used to analyze the percentage of free amino groups in gelatin that reacted with ADA. A colored ninhydrin chromophore was produced while crosslinking hydrogels of 1 g were heated in a water bath for 20 min at 100 °C with 2% *w*/*v* ninhydrin solution. A UV spectrophotometer (UV-Vis spectrophotometer 117 (1 NM) Gujarat (India)) was used to measure the solution’s optical absorbance at 570 nm; after it had cooled to room temperature, the number of free amino groups in the crosslinked hydrogels that reacted with ninhydrin after heating was proportional to the solution’s optical absorbances. A standard curve of glycine was used to determine the concentration of free amino groups in the crosslinked hydrogels. The following equation was used to calculate the degree of crosslinking [36]

Degree of crosslinking (%) = [(NH_2_) _NC_ − (NH2) _C_]/(NH_2_) _NC_)] × 100 (2)

where (NH_2_) _NC_ and (NH_2_) _C_ are the mole fractions of non-crosslinked, free amino groups.

#### 5.1.2. FTIR Spectroscopy

FT-IR spectra of alginate, ADA, gelatin, PEG, ADA + GEL, and hydrogel were measured using PerkinElmer Spectrum IR Version 10.7.2.

#### 5.1.3. Studies on the Interaction of ADA and Borate

^11^B NMR (boron nuclear magnetic resonance) spectra of ADA with 0.1 M borax, 0.1 M borax alone, and 0.1 M boric acid were recorded using Ecz- 400 Jeol (JOEL Ltd., Tokyo, Japan) for the solid state for ^11^B nuclei at 128 MHz [26].

#### 5.1.4. Gelation Temperature and Gelling Time Measurements

The gelation temperature and gelling time were measured at various temperatures (25°, 30°, 37 °C). A 10 mL glass beaker was magnetically stirred with 1 mL of 7% ADA + 0.1 M Borax + 8% GEL + 1% PEG solution using a Teflon-coated stir bead of 10 mm length and 5 mm diameter. The duration of the stir bar’s stop was recorded as the gelling time. All experiments were conducted in triplicate gelation time and investigated through rheology analysis at 37 °C [54,71].

#### 5.1.5. Rheological Property

The Modular Compact Rheometer MCR 102 (Anton Paar, India Pvt Ltd., Gurugram, India) was used to measure the rheological characteristics at 37 °C. Rheoplus/32 software (version V3.612006273-33024) was used with parallel-plate geometry. The gelation time was determined via time sweep rheology analysis. To do this, 1 mL of 70% ADA+ 0.1 M borax + gelatin + PEG was rapidly combined and put on the rheometer’s lower plate. The experiment was conducted at an angular frequency of 1 rad s^−1^ and 10% strain [36].

#### 5.1.6. Swelling Behavior of Hydrogel

The gravimetric method was employed to evaluate hydrogel water absorption. The swelling behavior of the hydrogel was carried out as follows: roughly 0.3 g (W_O_) of hydrogel was weighed and submersed in 10 mL of PBS at 37 °C for 24 h to reach equilibrium swelling. The swollen hydrogel was weighed after removing the buoyant with filter paper (Ws). The following equation expresses the swelling ratio (SR) [51]:

Swelling ratio (%) = ***W***_***s***_ − ***W***_***o***_/***W***_***o***_ × **100** (3)

where SR is the swelling ratio.

#### 5.1.7. Mechanical Property

The mechanical behavior was tested by compression tests using a universal testing machine model (Zwick Roell) instrument. Firstly, we prepared cylindrical hydrogels (about Ǿ15 mm × 4 mm) and allowed it to swell at a temperature of 37 °C until equilibrium was reached; then, we put the hydrogel on the lower plate of the instrument and compressed it using the upper plate at a strain/speed of 5 mm/min after recording the diameter and thickness of the hydrogels with the corresponding displacement set at 80%. All samples were analyzed in triplicate to obtain the stress–strain curve [52,72].

#### 5.1.8. Degradation

The degradation of biomaterials is an essential phenomenon for tissue engineering applications. Throughout 10, 60, and 100 days, the in vitro degradation of gels was monitored. Following hydrogel synthesis, 0.3 g of hydrogel was weighed as (W0) and submerged in 10 mL of PBS that was incubated at 37 °C with weekly changes of the buffer. The hydrogels were then gently removed from the PBS medium, and excess water from the surface was removed using filter paper. After that, the hydrogels were weighed (Wt). Weight loss was monitored. The following formula was used to calculate each period’s weight loss percentage (ΔW %) [42,73].

(4) ∆W(%)=(W0−Wt)/W0×100%

#### 5.1.9. Morphology

Scanning electron microscopy was used to analyze the morphology of hydrogels. The samples were mounted on the aluminum holder stubs using double-sticky carbon tape and coated with Au/Pu in an SPI-MODULETM high-resolution sputter coater, examined in a ZEISS scanning microscope at 15 kV.

### 5.2. Syringeability

The injectability value was defined as the time required to expel 1 mL of the hydrogel. A precise 18-gauge needle was utilized to evaluate the sensitivity of hydrogels. Before measurement, all syringes were equilibrated for 10 min at 25 °C, during which any air bubbles were carefully eliminated until a small amount of the hydrogel solution emerged. Subsequently, each hydrogel solution was smoothly passed through the syringe at 25 °C, and the ease of injection was visualized. All measurements were tested in triplicate [42,71].

### 5.3. Statistical Analysis

The results are presented as the mean ± standard deviation, and each experimental sample was replicated at least three times. All statistical analyses were conducted using one-way ANOVA using JMP version 17 software. All showed a confidence level of satisfactory significant difference (*p* < 0.05).

## Figures and Tables

**Table 1:** Summary of results of degree of oxidation of ADA obtained by periodate oxidation of sodium alginate at different periodate concentrations with its obtained yield.

Periodate Equivalent	Degree of Oxidation (%)	Yield (%)
60%	54	50
80%	70	70

**Table 2:** The parameters of the GPC analysis for molecular weight determination.

Peak No	Retention Time RT (min)	Mw (g/mol)	Mn (g/mol)	PD
Sodium alginate				
1	12.557	175,100	1,396,201	1.25
2	16.596	30,999	25,350	1.22
3	18.805	2549	2123	1.20
Alginate di-aldehyde (54%)				
1	14.96	156,290	131,512	1.18
2	16.945	22,859	19,749	1.15
3	17.425	11,494	10,010	1.14
4	19.160	1782	1578	1.12
Alginate di-aldehyde (70%)				
1	15.023	161,733	140,539	1.06
2	16.979	22,859	22,072	1.03
3	17.498	9891	1529	1.02
4	19.243	1620	1591	1.01

**Table 3:** Viscosity of sodium alginate and ADA.

Sample Value	Viscosity (cps)
Sodium alginate	80.000 *
54% ADA	34.000 *
70% ADA	9.300 *

* Represents a significant difference.

**Table 4:** Degree of crosslinking of hydrogel.

Formula	% Degree of Crosslinking
A1	52.56 ± 2
A2	62.56 ± 3

A significant difference was represented (*p* < 0.05).

**Table 5:** Summary of results of gelling time and gelling temperature obtained by magnetic stirrer method for formulated hydrogel with different borax concentrations.

Hydrogel (1 mL)	Gelation Temperature	Gelation Time in Minutes
A2 + 0.1 M Borax	25 ℃	No gelation
A2 + 0.1 M Borax	30 ℃	No gelation
A2 + 0.1 M Borax	37 ℃	2.5
A2 + 0.05 MBorax	37 ℃	8
A1 + 0.1 M Borax	25 ℃	No gelation
A1 + 0.1 M Borax	30 ℃	No gelation
A1 + 0.1 M Borax	37 ℃	4.5
A1 + 0.05 M Borax	37 ℃	12

**Table 6:** Swelling ratio (%) in PBS at 37 °C for hydrogels.

Hydrogel	Swelling Ratio
A1	580
A2	966

**Table 7:** Injection time of A1 and A2 hydrogels.

Gel	Time (s)
A1 hydrogel	276
A2 hydrogel	212

**Table 8:** Composition of hydrogel [42].

Sample Label	The Concentration of Polymers in Solutions (*w*/*v* %)			
	ADA	PEG	GEL	Borax
(54%)ADA/PEG/GEL hydrogel (A1)	7	1	7	0.1 M
(70%) ADA + PEG + GEL hydrogel (A2)	7	1	9	0.1 M

ADA—alginate di-aldehyde; PEG—polyethylene glycol; GEL—gelatin.

## Data Availability

Data are contained within the article.

## References

[1] Kuo C.K., Ma P.X. (2001). Ionically Crosslinked Alginate Hydrogels as Scaffolds for Tissue Engineering: Part 1. Structure, Gelation Rate and Mechanical Properties. Biomaterials.

[2] Vega S.L., Kwon M.Y., Burdick J.A. (2017). Recent Advances in Hydrogels for Cartilage Tissue Engineering. Eur. Cell Mater..

[3] Chen F.-M., Liu X. (2016). Advancing Biomaterials of Human Origin for Tissue Engineering. Prog. Polym. Sci..

[4] Hou Q., Bank P.A.D., Shakesheff K.M. (2004). Injectable Scaffolds for Tissue Regeneration. J. Mater. Chem..

[5] Ahmed E.M. (2015). Hydrogel: Preparation, Characterization, and Applications: A Review. J. Adv. Res..

[6] Tessmar J.K., Göpferich A.M. (2007). Matrices and Scaffolds for Protein Delivery in Tissue Engineering. Adv. Drug Deliv. Rev..

[7] Packhaeuser C.B., Schnieders J., Oster C.G., Kissel T. (2004). In Situ Forming Parenteral Drug Delivery Systems: An Overview. Eur. J. Pharm. Biopharm..

[8] Kurisawa M., Chung J.E., Yang Y.Y., Gao S.J., Uyama H. (2005). Injectable Biodegradable Hydrogels Composed of Hyaluronic Acid–Tyramine Conjugates for Drug Delivery and Tissue Engineering. Chem. Commun..

[9] Zheng Shu X., Liu Y., Palumbo F.S., Luo Y., Prestwich G.D. (2004). In Situ Crosslinkable Hyaluronan Hydrogels for Tissue Engineering. Biomaterials.

[10] Nguyen M.K., Lee D.S. (2010). Injectable Biodegradable Hydrogels. Macromol. Biosci..

[11] Khorshidi S., Karkhaneh A. (2016). A Self-Crosslinking Tri-Component Hydrogel Based on Functionalized Polysaccharides and Gelatin for Tissue Engineering Applications. Mater. Lett..

[12] Cascone S., Lamberti G. (2020). Hydrogel-Based Commercial Products for Biomedical Applications: A Review. Int. J. Pharm..

[13] Fertah M., Venkatesan J., Anil S., Kim S.-K. (2017). Chapter 2—Isolation and Characterization of Alginate from Seaweed. Seaweed Polysaccharides.

[14] Hasnain M.S., Nayak A.K. (2019). Alginates: Versatile Polymers in Biomedical Applications and Therapeutics.

[15] Moradali M.F., Ghods S., Rehm B.H.A., Rehm B.H.A., Moradali M.F. (2018). Alginate Biosynthesis and Biotechnological Production. Alginates and Their Biomedical Applications.

[16] Rosiak P., Latanska I., Paul P., Sujka W., Kolesinska B. (2021). Modification of Alginates to Modulate Their Physic-Chemical Properties and Obtain Biomaterials with Different Functional Properties. Molecules.

[17] Alginate as Immobilization Matrix for Cells: Trends in Biotechnology. https://www.cell.com/trends/biotechnology/abstract/0167-7799(90)90139-O.

[18] Draget K.I., Skjåk-Bræk G., Smidsrød O. (1997). Alginate Based New Materials. Int. J. Biol. Macromol..

[19] Lee K.Y., Mooney D.J. (2012). Alginate: Properties and Biomedical Applications. Prog. Polym. Sci..

[20] Hernández-González A.C., Téllez-Jurado L., Rodríguez-Lorenzo L.M. (2020). Alginate Hydrogels for Bone Tissue Engineering, from Injectables to Bioprinting: A Review. Carbohydr. Polym..

[21] Gao C., Liu M., Chen J., Zhang X. (2009). Preparation and Controlled Degradation of Oxidized Sodium Alginate Hydrogel. Polym. Degrad. Stab..

[22] Wang H., Chen X., Wen Y., Li D., Sun X., Liu Z., Yan H., Lin Q. (2022). A Study on the Correlation between the Oxidation Degree of Oxidized Sodium Alginate on Its Degradability and Gelation. Polymers.

[23] Reakasame S., Boccaccini A.R. (2018). Oxidized Alginate-Based Hydrogels for Tissue Engineering Applications: A Review. Biomacromolecules.

[24] Szekalska M., Puciłowska A., Szymańska E., Ciosek P., Winnicka K. (2016). Alginate: Current Use and Future Perspectives in Pharmaceutical and Biomedical Applications. Int. J. Polym. Sci..

[25] Kuijpers A.J., Engbers G.H.M., Krijgsveld J., Zaat S.A.J., Dankert J., Feijen J. (2000). Cross-Linking and Characterisation of Gelatin Matrices for Biomedical Applications. J. Biomater. Sci. Polym. Ed..

[26] Balakrishnan B., Jayakrishnan A. (2005). Self-Cross-Linking Biopolymers as Injectable in Situ Forming Biodegradable Scaffolds. Biomaterials.

[27] Sweet D.V., Anderson V.P., Fang J.C.F. (1999). An Overview of the Registry of Toxic Effects of Chemical Substances (RTECS): Critical Information on Chemical Hazards. Chem. Health Saf..

[28] Jansen J.A., Andersen J., Schou J.S. (1984). Boric Acid Single Dose Pharmacokinetics after Intravenous Administration to Man. Arch Toxicol.

[29] Su J., Satchell S.C., Wertheim J.A., Shah R.N. (2019). Poly(Ethylene Glycol)-Crosslinked Gelatin Hydrogel Substrates with Conjugated Bioactive Peptides Influence Endothelial Cell Behavior. Biomaterials.

[30] Bigi A., Panzavolta S., Rubini K. (2004). Relationship between Triple-Helix Content and Mechanical Properties of Gelatin Films. Biomaterials.

[31] Tissue Engineering Strategies for Intervertebral Disc Treatment Using Functional Polymers. https://www.mdpi.com/2073-4360/11/5/872.

[32] Mellati A., Hasanzadeh E., Gholipourmalekabadi M., Enderami S.E. (2021). Injectable Nanocomposite Hydrogels as an Emerging Platform for Biomedical Applications: A Review. Mater. Sci. Eng. C.

[33] Boanini E., Rubini K., Panzavolta S., Bigi A. (2010). Chemico-Physical Characterization of Gelatin Films Modified with Oxidized Alginate. Acta Biomater..

[34] Zhang H., Cheng J., Ao Q. (2021). Preparation of Alginate-Based Biomaterials and Their Applications in Biomedicine. Mar. Drugs.

[35] Pawar S.N., Edgar K.J. (2012). Alginate Derivatization: A Review of Chemistry, Properties and Applications. Biomaterials.

[36] Resmi R., Parvathy J., John A., Joseph R. (2020). Injectable Self-Crosslinking Hydrogels for Meniscal Repair: A Study with Oxidized Alginate and Gelatin. Carbohydr. Polym..

[37] Gomez C.G., Rinaudo M., Villar M.A. (2007). Oxidation of Sodium Alginate and Characterization of the Oxidized Derivatives. Carbohydr. Polym..

[38] Nonsuwan P., Matsugami A., Hayashi F., Hyon S.-H., Matsumura K. (2019). Controlling the Degradation of an Oxidized Dextran-Based Hydrogel Independent of the Mechanical Properties. Carbohydr. Polym..

[39] van Haveren J., van den Burg M.H.B., Peters J.A., Batelaan J.G., Kieboom A.P.G., van Bekkum H. (1991). Structure and Stability, as a Function of pH, of Borate Esters of Carbohydrate Oximes and Related Compounds in Aqueous Media Studied by 11B and 13C NMR Spectroscopy. J. Chem. Soc., Perkin Trans..

[40] Emami Z., Ehsani M., Zandi M., Foudazi R. (2018). Controlling Alginate Oxidation Conditions for Making Alginate-Gelatin Hydrogels. Carbohydr. Polym..

[41] Ding W., Zhou J., Zeng Y., Wang Y., Shi B. (2017). Preparation of Oxidized Sodium Alginate with Different Molecular Weights and Its Application for Crosslinking Collagen Fiber. Carbohydr. Polym..

[42] Naghizadeh Z., Karkhaneh A., Khojasteh A. (2018). Self-Crosslinking Effect of Chitosan and Gelatin on Alginate Based Hydrogels: Injectable in Situ Forming Scaffolds. Mater. Sci. Eng. C.

[43] Bhattarai N., Edmondson D., Veiseh O., Matsen F.A. (2005). Evaluation of an in Situ Forming Hydrogel Wound Dressing Based on Oxidized Alginate and Gelatin. Biomaterials.

[44] Hunt C.D., Idso J.P. (1999). Dietary Boron as a Physiological Regulator of the Normal Inflammatory Response: A Review and Current Research Progress. J. Trace Elem. Exp. Med..

[45] Shen K.-H., Chiu T.-H., Teng K.-C., Yu J., Yeh Y.-C. (2023). Fabrication of Triple-Crosslinked Gelatin/Alginate Hydrogels for Controlled Release Applications. Int. J. Biol. Macromol..

[46] Bishop M., Shahid N., Yang J., Barron A.R. (2004). Determination of the Mode and Efficacy of the Cross-Linking of Guar by Borate Using MAS 11B NMR of Borate Cross-Linked Guar in Combination with Solution 11B NMR of Model Systems. Dalton Trans..

[47] Shao C., Miyazaki Y., Matsuoka S., Yoshimura K., Sakashita H. (2000). Complexation of Borate with Cross-Linked Polysaccharide Anion Exchanger:  11B NMR and Adsorption Properties Studies. Macromolecules.

[48] Artzi N., Oliva N., Puron C., Shitreet S., Artzi S., bon Ramos A., Groothuis A., Sahagian G., Edelman E.R. (2011). In Vivo and in Vitro Tracking of Erosion in Biodegradable Materials Using Non-Invasive Fluorescence Imaging. Nat. Mater..

[49] Sarker B., Papageorgiou D.G., Silva R., Zehnder T., Gul-E-Noor F., Bertmer M., Kaschta J., Chrissafis K., Detsch R., Boccaccini A.R. (2014). Fabrication of Alginate–Gelatin Crosslinked Hydrogel Microcapsules and Evaluation of the Microstructure and Physico-Chemical Properties. J. Mater. Chem. B.

[50] Ketabat F., Karkhaneh A., Aghdam R.M., Tafti S.H.A. (2017). Injectable Conductive Collagen/Alginate/Polypyrrole Hydrogels as a Biocompatible System for Biomedical Applications. J. Biomater. Sci. Polym. Ed..

[51] Ghanbari M., Salavati-Niasari M., Mohandes F. (2021). Injectable Hydrogels Based on Oxidized Alginate-Gelatin Reinforced by Carbon Nitride Quantum Dots for Tissue Engineering. Int. J. Pharm..

[52] Sivan S.S., Roberts S., Urban J.P.G., Menage J., Bramhill J., Campbell D., Franklin V.J., Lydon F., Merkher Y., Maroudas A. (2014). Injectable Hydrogels with High Fixed Charge Density and Swelling Pressure for Nucleus Pulposus Repair: Biomimetic Glycosaminoglycan Analogues. Acta Biomater..

[53] Stojkov G., Niyazov Z., Picchioni F., Bose R.K. (2021). Relationship between Structure and Rheology of Hydrogels for Various Applications. Gels.

[54] Yuan L., Wu Y., Fang J., Wei X., Gu Q., El-Hamshary H., Al-Deyab S.S., Morsi Y., Mo X. (2017). Modified Alginate and Gelatin Cross-Linked Hydrogels for Soft Tissue Adhesive. Artif. Cells Nanomed. Biotechnol..

[55] Cai K., Zhang J., Deng L., Yang L., Hu Y., Chen C., Xue L., Wang L. (2007). Physical and Biological Properties of a Novel Hydrogel Composite Based on Oxidized Alginate, Gelatin and Tricalcium Phosphate for Bone Tissue Engineering. Adv. Eng. Mater..

[56] (2012). Fabrication of Oxidized Alginate-Gelatin-BCP Hydrogels and Evaluation of the Microstructure, Material Properties and Biocompatibility for Bone Tissue Regeneration—Thi-Phuong Nguyen, Byong-Taek Lee. https://journals.sagepub.com/doi/abs/10.1177/0885328211404265.

[57] Stahl P.J., Romano N.H., Wirtz D., Yu S.M. (2010). PEG-Based Hydrogels with Collagen Mimetic Peptide-Mediated and Tunable Physical Cross-Links. Biomacromolecules.

[58] Lin C.-C., Anseth K.S. (2009). PEG Hydrogels for the Controlled Release of Biomolecules in Regenerative Medicine. Pharm. Res..

[59] Narayanaswamy R., Torchilin V.P. (2019). Hydrogels and Their Applications in Targeted Drug Delivery. The Road from Nanomedicine to Precision Medicine.

[60] Drury J.L., Mooney D.J. (2003). Hydrogels for Tissue Engineering: Scaffold Design Variables and Applications. Biomaterials.

[61] Bouhadir K.H., Lee K.Y., Alsberg E., Damm K.L., Anderson K.W., Mooney D.J. (2001). Degradation of Partially Oxidized Alginate and Its Potential Application for Tissue Engineering. Biotechnol. Prog..

[62] Teng K., An Q., Chen Y., Zhang Y., Zhao Y. (2021). Recent Development of Alginate-Based Materials and Their Versatile Functions in Biomedicine, Flexible Electronics, and Environmental Uses. ACS Biomater. Sci. Eng..

[63] Wendt D., Jakob M., Martin I. (2005). Bioreactor-Based Engineering of Osteochondral Grafts: From Model Systems to Tissue Manufacturing. J. Biosci. Bioeng..

[64] Kong H.J., Mooney D.J. (2003). The Effects of Poly(Ethyleneimine) (PEI) Molecular Weight on Reinforcement of Alginate Hydrogels. Cell Transpl..

[65] Hu X., Liang R., Li J., Liu Z., Sun G. (2019). Mechanically Strong Hydrogels Achieved by Designing Homogeneous Network Structure. Mater. Des..

[66] Radhakrishnan A., Jose G.M., Kurup M. (2015). PEG-Penetrated Chitosan–Alginate Co-Polysaccharide-Based Partially and Fully Cross-Linked Hydrogels as ECM Mimic for Tissue Engineering Applications. Prog. Biomater..

[67] Bružauskaitė I., Bironaitė D., Bagdonas E., Bernotienė E. (2016). Scaffolds and Cells for Tissue Regeneration: Different Scaffold Pore Sizes—Different Cell Effects. Cytotechnology.

[68] Coluccino L., Gottardi R., Ayadi F., Athanassiou A., Tuan R.S., Ceseracciu L. (2018). Porous Poly(Vinyl Alcohol)-Based Hydrogel for Knee Meniscus Functional Repair. ACS Biomater. Sci. Eng..

[69] Capanema N.S.V., Mansur A.A.P., de Jesus A.C., Carvalho S.M., de Oliveira L.C., Mansur H.S. (2018). Superabsorbent Crosslinked Carboxymethyl Cellulose-PEG Hydrogels for Potential Wound Dressing Applications. Int. J. Biol. Macromol..

[70] Balakrishnan B., Lesieur S., Labarre D., Jayakrishnan A. (2005). Periodate Oxidation of Sodium Alginate in Water and in Ethanol–Water Mixture: A Comparative Study. Carbohydr. Res..

[71] Sengupta B., Sharma V.P., Udayabhanu G. (2012). Gelation Studies of an Organically Cross-Linked Polyacrylamide Water Shut-off Gel System at Different Temperatures and pH. J. Pet. Sci. Eng..

[72] Yuan L., Wu Y., Gu Q., El-Hamshary H., El-Newehy M., Mo X. (2017). Injectable Photo Crosslinked Enhanced Double-Network Hydrogels from Modified Sodium Alginate and Gelatin. Int. J. Biol. Macromol..

[73] Frith J.E., Cameron A.R., Menzies D.J., Ghosh P., Whitehead D.L., Gronthos S., Zannettino A.C.W., Cooper-White J.J. (2013). An Injectable Hydrogel Incorporating Mesenchymal Precursor Cells and Pentosan Polysulphate for Intervertebral Disc Regeneration. Biomaterials.

